# Extreme enrichment of VNTR-associated polymorphicity in human subtelomeres: genes with most VNTRs are predominantly expressed in the brain

**DOI:** 10.1038/s41398-020-01060-5

**Published:** 2020-11-02

**Authors:** Jasper Linthorst, Wim Meert, Matthew S. Hestand, Jonas Korlach, Joris Robert Vermeesch, Marcel J. T. Reinders, Henne Holstege

**Affiliations:** 1 Department of Clinical Genetics, Amsterdam Neuroscience, Vrije Universiteit Amsterdam, Amsterdam UMC, Amsterdam, The Netherlands; 2 Delft Bioinformatics Lab, Delft University of Technology, Delft, The Netherlands; 3 Department of Human Genetics, KU Leuven, Leuven, Belgium; 4 Pacific Biosciences, Menlo Park, CA USA; 5 Alzheimer Center Amsterdam, Department of Neurology, Amsterdam Neuroscience, Vrije Universiteit Amsterdam, Amsterdam UMC, Amsterdam, The Netherlands

**Keywords:** Clinical genetics, Clinical genetics

## Abstract

The human genome harbors numerous structural variants (SVs) which, due to their repetitive nature, are currently underexplored in short-read whole-genome sequencing approaches. Using single-molecule, real-time (SMRT) long-read sequencing technology in combination with FALCON-Unzip, we generated a de novo assembly of the diploid genome of a 115-year-old Dutch cognitively healthy woman. We combined this assembly with two previously published haploid assemblies (CHM1 and CHM13) and the GRCh38 reference genome to create a compendium of SVs that occur across five independent human haplotypes using the graph-based multi-genome aligner REVEAL. Across these five haplotypes, we detected 31,680 euchromatic SVs (>50 bp). Of these, ~62% were comprised of repetitive sequences with ‘variable number tandem repeats’ (VNTRs), ~10% were mobile elements (Alu, L1, and SVA), while the remaining variants were inversions and indels. We observed that VNTRs with GC-content >60% and repeat patterns longer than 15 bp were 21-fold enriched in the subtelomeric regions (within 5 Mb of the ends of chromosome arms). VNTR lengths can expand to exceed a critical length which is associated with impaired gene transcription. The genes that contained most VNTRs, of which PTPRN2 and DLGAP2 are the most prominent examples, were found to be predominantly expressed in the brain and associated with a wide variety of neurological disorders. Repeat-induced variation represents a sizeable fraction of the genetic variation in human genomes and should be included in investigations of genetic factors associated with phenotypic traits, specifically those associated with neurological disorders. We make available the long and short-read sequence data of the supercentenarian genome, and a compendium of SVs as identified across 5 human haplotypes.

## Background

Repetitive sequences give rise to a myriad of structural variants (SVs), and recent findings indicate that these might explain at least part of the missing heritability for many traits^1–5^. Repeat sequences have been associated with pathogenicity, as they were shown to underly several diseases, many of which affect the nervous system^6–8^. A common characteristic of familial repeat-associated nervous system diseases is the increased disease severity and decreased age of disease-onset in successive generations, termed ‘genetic anticipation’. This phenomenon follows from the dynamic nature of repeat variants, as repeat sequences can expand with each generation. Once a repeat sequence exceeds a critical length, this may lead to impaired gene transcription, the effect of which may be further aggravated upon further expansion in the next generation. Genetic anticipation is a characteristic for neurological diseases such as Huntington’s disease, amyotrophic lateral sclerosis (ALS), schizophrenia, and bipolar disorder^9^. A recent report indicated that when the 25 nt subunit repeat sequence in the *ABCA7* gene expands to exceed ~5200 nt, this associates with a ~4.5-fold increased risk for Alzheimer’s disease^5^.

The genome holds many polymorphic regions, but very little is known about the effects these may have on disease risk. Until recently, investigating the impact of SVs on the risk of having a trait was challenging. Using common sequencing approaches, the assessment of large repetitive regions is difficult because short 100–150 bp sequence-reads do not span the entire structural variant^10^. The solution to this problem is to generate longer sequencing reads. Various studies have shown that PacBio’s single-molecule, real-time (SMRT) long-read sequencing can be used to reveal large numbers of novel SVs in previously inaccessible regions of the human genome^10–14^.

To study the highly polymorphic repetitive loci in a genome-wide manner, we set out to compare the SVs identified in five haplotypes. First, we generated SMRT long-read sequences of the diploid genome of a Dutch woman who reached 115 years while remaining cognitively healthy (W115, see Box 1)^15^. We then compared the two haplotypes of the diploid W115 genome assembly with two publicly available haploid genome assemblies^12^ and the GRCh38 reference assembly^16^. While previous work on detecting SVs from long-read sequencing data used haploid human cell lines (complete hydatidiform mole DNA)^10,12,17^, actual human genomes are diploid. This diploidy complicates the accurate detection of structural genomic variation. A further complication is the repetitive nature of most SVs: since there are many ways in which two repetitive sequences can be aligned, this leads to inherent uncertainties in the positioning of SVs^18^. This becomes especially problematic when calling SVs across multiple sequences. Here, we used the graph-based multi-genome aligner REVEAL^19^ to simultaneously align multiple sequences in a graph and threshold the alignment uncertainty to obtain consistently positioned SV calls across multiple genome assemblies.

Here, we present and interpret a compendium of the structural variants identified across the five investigated haplotypes. Furthermore, we release the raw long-read and short-read sequencing data of the W115 supercentenarian, a genome which is likely depleted with genetic elements associated with increased disease risk and enriched with elements that have protective effect^20^. We reason that the lengths and sequences of the SVs in the W115 genome are representative of non-pathogenic SVs, which represents a useful resource for genetics research.

BOX 1 W115: Hendrikje van Andel-Schipper (1890–2005), reached 115 years without cognitive decline Hendrikje Schipper was born in 1890, in the small village of Smilde, in the province of Drenthe in the North of the Netherlands. At the time of her death, she was aged 115 years and 62 days. Her mother (1862–1963) died at 101 years in full cognitive health. Her father (1864–1950) died at the age of 85. Aged 111 she said in a newspaper interview: “During the last five years I have not stayed in bed for one day. My adage is: I refuse to become petulant”^83^. She had a great-nephew who reached 106 years, who also remained active until a very high age, with retained cognitive health.

Hendrikje Schipper was born prematurely; she weighed <1400 g (3 lbs 1 oz) and was not expected to survive. She was a sickly child and was removed from school on advice of a local doctor. Her father, headmaster at the local school, taught her to read and write. In 1939, Miss Schipper married Dick van Andel at the age of 47, taking the hyphenated name of Van Andel-Schipper, which is customary in the Netherlands. The couple remained childless. During the Second World War, she and her husband moved to the town of Hoogeveen, also in the province of Drenthe, where she lived during the remainder of her life. Her husband died from esophagus cancer in 1959. In 1972, at the age of 82, Mrs van Andel-Schipper donated her body to science^84^, and her body was to be moved to the University of Groningen upon her death. At age 100, Mrs van Andel was diagnosed with breast cancer and underwent a mastectomy^15^. She continued to live on her own before moving into a retirement home at the age of 105, due to bad eyesight. At the age of 112, she asked the director of the retirement home to contact the hospital in Groningen to inquire whether donating her (frail) body to science was still of value. This led to a visit by researchers who subjected her to a neuropsychological testing battery at the ages of 112 and 113, which indicated that her brain was fully functional^85^. Furthermore, post-mortem investigation of her body indicated that she died from a large gastric tumor. Intriguingly, she had no atherosclerosis throughout her body, and her brain showed little tau pathology (Braak Stage II), nor did she have any amyloid plaques (Thal Stage 0). *Of note: all direct family members known to be alive at the start of this project in 2014, whose genomes overlap ~12.5% or more with Mrs. van Andel-Schipper’s genome, provided written consent for revealing her full name and history, asserting that this project is in line with her spirit and her long-term wish to contribute to science*.

## Methods

### W115 DNA isolation

Heart tissue was snap frozen several hours after the death of W115 and kept at −80 °C. DNA from a frozen whole blood sample was isolated with the Promega Wizard Kit. Inspection on gel indicated that DNA from all tissues was high-molecular-weight genomic DNA. We sequenced genomic DNA across three tissues across 269 SMRT Cells (Table S1) on a PacBio RSII sequencer, which generated 16,411,063 reads larger than 500 bp with an average read length of 8334 bp. This equalled an estimate of ~45× genome-wide coverage. We also generated 100× coverage with 150 bp paired-end Illumina sequencing for the W115 genome and used BWA-mem^21^ (version 0.7.17-r1188 with default settings) and GATK^22^ (HaplotypeCaller version 3.8 with default settings) to detect SVs.

### Haplotype assembly W115

We used FALCON-Unzip to assemble the SMRT sequencing data into 2729 partially phased contigs (2729 primary contigs with a cumulative size of 2.82 GB and 24,122 associated haplotype phased contigs with a cumulative size of 1.44 GB) with an N50 of ~7 Mb and a total reconstructed genome size of about 2.82 Gb. From this data, we constructed two pseudo-haplotypes (Fig. S1) that represent the two homologous chromosomes of the W115 genome.

### CHM1 and CHM13 haplotypes

Publicly available SMRT sequencing assemblies for the haploid CHM1 and CHM13 cell lines were downloaded from GenBank, using accession id’s GCA_001297185.1 and GCA_000983455.2, respectively (Table 1).

**Table 1:** Characteristics of the genome assemblies utilized in this study.

Assembly	Ploidy	Assembler	N50	Assembly accession
GRCh38	N/A	N/A	57.9 Mb	GCA_000001405.28
GCA_001297185.1 (CHM1)	Haploid	FALCON	26.9 Mb	GCA_001297185.1
GCA_000983455.2 (CHM13)	Haploid	FALCON	10.5 Mb	GCA_000983455.2
W115	Diploid	FALCON-Unzip	6.8 Mb	Haplotype 1: GCA_903995575 Haplotype 2: GCA_904060995

Note that the assemblies have varying levels of contiguity.

### Global alignment with rearrangements

The REVEAL package^19^ was used to align the five haplotypes into a graph-based representation. *REVEAL-transform* was used to model rearrangements (as well as assembly errors, which from the perspective of the assembly, manifest in the same way) between the SMRT sequencing assemblies and the GRCh38 reference assembly in a breakpoint graph. Then, the colinear layouts of the five resulting pseudo-chromosomes (one for each haplotype) were simultaneously anchored using *REVEAL-rem*. All resulting variants (bubbles), that did not exceed 10,000 bp in size (predefined) were then aligned using a probabilistic consistency-based multiple sequence alignment using the ProbCons algorithm^23^. Note that we slightly adapted the ProbCons algorithm to (1) reflect optimal state emission and transition probabilities (as was previously shown^24^), for multiple nucleotide sequence alignment, and (2) we adapted the consistency transformation such that also the confidence of columns in the multiple sequence alignment that involved gaps could be computed. Finally, only highly confident columns (≥99%) in the multiple sequence alignments were merged as nodes into the final graph. This way, SNVs, short indels as well as large repeat-induced variants and rearrangements are encoded into a single graph data structure, with explicit node boundaries determined by the confidence of the multiple sequence alignments.

### Structural variant characterization

As most SVs are multi-allelic, we defined a structural variant as a locus for which the largest allele is at least 50 bp longer than the shortest allele. An SV was considered ‘unique’, when the difference in allele sizes amongst the other four haplotypes was less than 50 bp.

#### Mobile element insertions (MEI)

To characterize the identified structural variants, we first inspected all alleles for the presence of known interspersed repeat elements^25^. When one of the variable alleles has an 80% reciprocal overlap with a known Interspersed Repeat Element, we define the variant as a Mobile Element Insertion (MEI).

#### Variable number of tandem repeats (VNTR)

Throughout literature, polymorphic tandem repeats are referred to in various ways. Depending on the size of the repeated pattern they are either called (mini, micro, macro)-satellites, short tandem repeats (STRs), variable number tandem repeats (VNTRs), simple sequence repeats (SSRs), and even copy-number variations (CNVs). Here we simply referred to these variants as VNTRs (note that throughout this paper we only consider variants > =50 bp). SVs that were not classified as a MEI were scanned using Tandem Repeat Finder^25^ using the following settings: “trf <seq> 2 7 7 80 10 20 500 -ngs -h”. Each allele was separately scanned for tandem repeat patterns. If the best scoring tandem repeat pattern on one of the 5 alleles spanned more than 50% of that allele, the SV was classified as a VNTR. To make sure we would also classify single unit insertions and deletions as VNTRs, we additionally intersected the list of SVs with the Tandem Repeat Finder annotations track on the GRCh38 reference assembly and also classified these SVs as VNTRs. The consensus size of the best scoring tandem repeat was further used for subsequent classification of the VNTR.

#### Rearrangements

Rearrangements change the order and orientation in which segments of the genome are organized. Therefore, as opposed to the colinear variants, we addressed these rearrangements with a pairwise comparison to the GRCh38 reference assembly. Inversions are balanced events (they do not lead to an increase or decrease of sequence) and are notoriously complicated to genotype. Furthermore, inversions often arise between large inverted repeat structures (low-copy repeats/segmental duplications), such that, in order to genotype them using sequencing data, reads need to first span these large repeat structures. Rearrangements were derived from an initial pairwise comparison to the GRCh38 reference implemented in *REVEAL-transform* (default parameters). This approach determines breakpoints between a target and a query assembly (given a set of cost parameters). As the SMRT assemblies used here are draft assemblies, we used these assemblies as the query while using GRCh38 as reference assembly. The result of this routine is a breakpoint graph (in GFA) in which all rearrangement breakpoints are modeled as edges. Apart from the graph, this routine also outputs a bed file with the order and orientation of parts of the query assembly with respect to the GRCh38 assembly. From these ordered and oriented segments, inversions were derived by extracting segments that are enclosed within larger segments in opposing orientation but consecutive on both reference and query contigs. Furthermore, all three segments are required to originate from the same contig, and the enclosing segments must be larger than the inverted segment.

#### Other

We often observed large regions of compound variant polymorphicity (e.g., combinations of multiple VNTRs and MEIs), large deletions of non-repetitive sequence, and known CNVs (essentially low-copy repeats for which the repeated pattern far exceeded the detection limits of Tandem Repeat Finder). Together, we classified these variants as ‘other’.

### Correlation between gene-VNTR-number and gene length with its expression in brain

We computed the correlation between the (1) length of a gene or (2) the number of VNTRs in that gene with the expression levels of that gene in the brain. For this, we determined the mean expression value across all brain-derived regions for each gene using the median gene-level TPM (transcripts per kilobase million) per brain-region as reported by the Genotype-Tissue Expression (GTEx) Project (file: GTEx_Analysis_2017-06-05_v8_RNASeQCv1.1.9_gene_tpm.gct.gz). We determined the correlation of the log10 of this mean expression value with gene length and VNTR number: the *spearmanr* function in the Scipy stats module was used to calculate correlation coefficients and *p*-values^26^.

## Results

### W115 de novo assembly

The haplotype-aware assembly^27^ of the W115 diploid genome (N50 = 6.8 Mb, see Table 1, and Fig. S1 for the assembly pipeline) resulted in two sets of 2729 contigs (one contig set per pseudo-haplotype) of which 1400 and 1395 could be uniquely positioned across, respectively, 1818 and 1837 distinct loci on the GRCh38 reference genome^16^ (see the “Methods” section). In total, we observed 1659 and 1680 gaps in the colinear alignment of the two haplotypes against the euchromatic part of the GRCh38 reference assembly (GRCh38; excluding charm, chrY, alt, and unplaced sequence and filtered by the following cytoband regions: acen, gvar, and stalk). These gaps resulted from rearrangements within a contig, lack of read overlap between consecutive contigs and un-spanned repetitive sequences. Of these gaps, ~62% coincided with known segmental duplications. Finally, with this assembly, we spanned ~94% of the euchromatic part of the GRCh38 assembly and span 96.7% of the protein-coding gene annotations (gencode v30).

### Identification of 31,680 large structural variants across five haplotypes

**Fig. 1:**
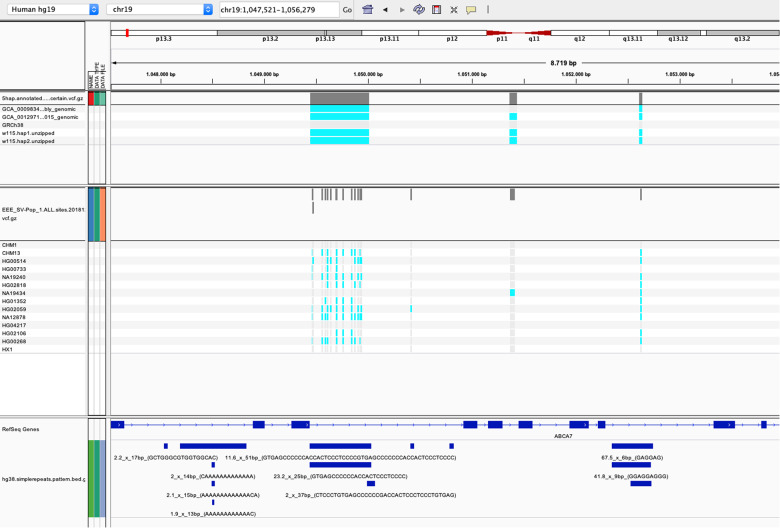
IGV screenshot of variant calls at the VNTR locus of the ABCA7 gene. TOP panel: the SV variant calls from REVEAL across five haplotypes. Boundaries of SV calls are determined by thresholding the certainty of a probabilistic consistency-based multiple sequence alignment. Middle panel: the SV variant calls from Audano et al.^28^. Bottom panel: the Tandem Repeat Finder annotations. The multi-allelic VNTR results in 14 different SVs with the method employed by Audano, while the SV call from REVEAL results in a single SV call, which aligns with the Tandem Repeat Finder annotation at that locus.

We then compared the colinear layouts of five assembled (pseudo) haplotypes by simultaneously aligning them in a graph and thresholding the alignment uncertainty to obtain consistently positioned SV calls (“Methods”): the W115 diploid genome, the GRCh38 reference assembly^16^ and the publicly available CHM1 and CHM13 haploid genome assemblies^12^ (Table 1). We detected a total of 31,680 colinear SVs in the euchromatic part of the human genome in which, across the five haplotypes, the shortest allele differed at least 50 bp from the largest allele (“Methods”). One example is a complex SV in the *ABCA7* locus, which, using our graph-based multi-genome alignment, we can detect as one consistent SV (Fig. 1).

**Fig. 2:**
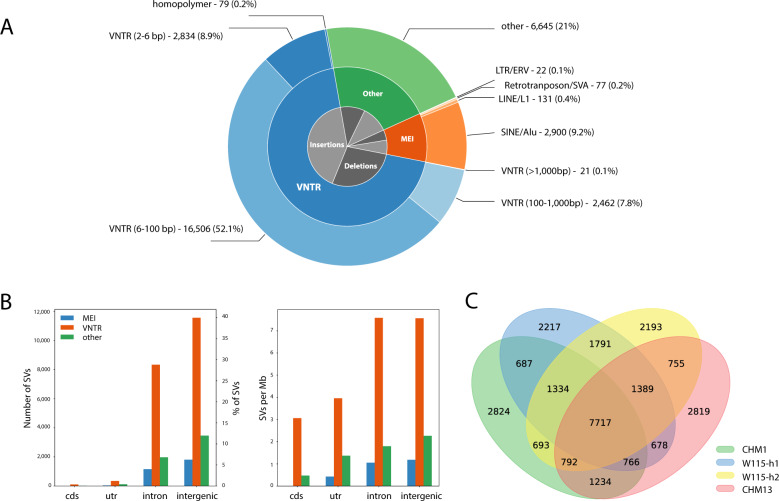
Characteristics of SVs across five human haplotypes. **A** Distribution of detected SVs across 5 human haplotypes classified by SV type. **B** Left panel: Counts of SVs per genomic region. Right panel: Number of SVs per Mb of assessable genomic sequence. **C** Overlap between SVs of the four non-reference haplotypes.

We then classified the detected SVs according to SV type (“Methods”) and found that 69.1% of all SVs are variable nucleotide tandem repeats (VNTRs) (Fig. 2A): 52.1% are VNTRs with a repeat pattern between 6 and 100 bp, and 7.8% has a repeat pattern between 100 and 1000 bp. Furthermore, 9.9% of all SVs are mobile element insertions (MEI), and 21% of all SVs were classified under ‘other’ SV types. Moreover, most SVs are intergenic (~58%) and intronic (~40%), while only a fraction of the SVs spans an untranslated region (1.7%) or coding region of a gene (0.4%) (Fig. 2B). Of these SVs, 2785 (9%) could not be spanned in the assembly of at least one of the five haplotypes. The resulting 28,922 SVs that could be assessed in all haplotypes were used for all subsequent analyses. We found that of these variations, 6909 (22%) were unique (“Methods”) to the W115 assembly.

### Unique and overlapping SVs within the GRCh38-based SV set

With current strategies, SVs from one genome are defined and annotated with respect to a reference genome. With REVEAL we simultaneously compare the SVs between five haplotypes, and then annotate with respect to one (here, the GRCh38 reference genome). As a result, our set of SVs also contains VNTRs with a contraction or expansion of >50 bp between two alleles from non-reference genomes; when the difference between each of the two alleles and the reference genome is <50 bp this SV will not be identified using other approaches. In total we detect 1033 such variants as SVs. To determine the overlap between the SV calls that could be assessed in all five haplotypes, with respect to the reference assembly, we excluded these 1033 loci, and obtained a set of 27,889 SVs. This set of SVs can be considered as a reference-based SV set. Using this GRCh38-based SV set, there are SVs that are unique for one of the non-reference haplotypes, as well as SVs for which all non-reference haplotypes have an allele that differs more than 50 bp from the reference allele. According to these definitions, we constructed a Venn diagram to represent this overlap (Fig. 2C). The four non-reference haplotypes on average have 2513 unique reference-based SVs (sd = 308), while 7717 reference-based SVs are observed in all four PacBio assemblies. Further, we see on average 973 (sd = 415) and 1070 (sd = 292) reference-based SVs shared between two and three haplotypes, respectively.

### SV comparison with a set of Audano et al

To further characterize our set of 28,922 detected SVs, we compared them with the SVs recently identified by Audano et al. across 15 human genomes^28^. Note that this is not trivial as boundaries of SVs shift between samples due to their repetitive and multi-allelic nature, which we solved by our graph-based multi-genome alignment. Audano et al used SMRT-SV to obtain 97,585 SV calls with respect to GRCh38 and then merged these (pairwise) calls based on a reciprocal overlap of 50%. This might result in multiple SVs, while we detect only one using our graph-based multi-genome alignment (Fig. 1). Therefore, to compare the two SV sets, we computed the ‘non-reciprocal overlap’ and found that 8145 of our SVs do not overlap with Audano’s SVs. Of the 6909 SVs that were ‘unique’ to our W115 assembly, 3093 SVs did not overlap with an SV in the Audano dataset (Table S2). The largest event that we detected in the genome of our W115 assembly, that was not previously reported by Audano et al. was a 56 kb homozygous deletion of the *BTNL8* gene^29^.

### Overlap with SVs obtained by short-read sequencing of W115 genome

To assess how many of these SVs could be identified using short-read sequencing, we compared the SVs identified in the W115 long-read assembly with respect to the GRCh38 reference and obtained a list of 22,611 SVs (here we did not exclude loci that could not be spanned in either one of the CHM assemblies). Using 150 bp paired-end Illumina short-read data derived from the W115 genome, we detected 5826 SVs, using a reference-based short-read variant calling approach (haplotype caller)^22^. We found that ~68% of these short-read based SVs (3950 of 5826 SVs) overlapped with the set of SVs that we obtained from our long-read assembly. Conversely, about ~83% (18,661 SVs) of all the 22,611 long-read based detected SVs were uniquely identified through long-read sequencing.

### SV enrichment in subtelomeres: driven by GC-rich VNTRs with long repeat patterns

**Fig. 3:**
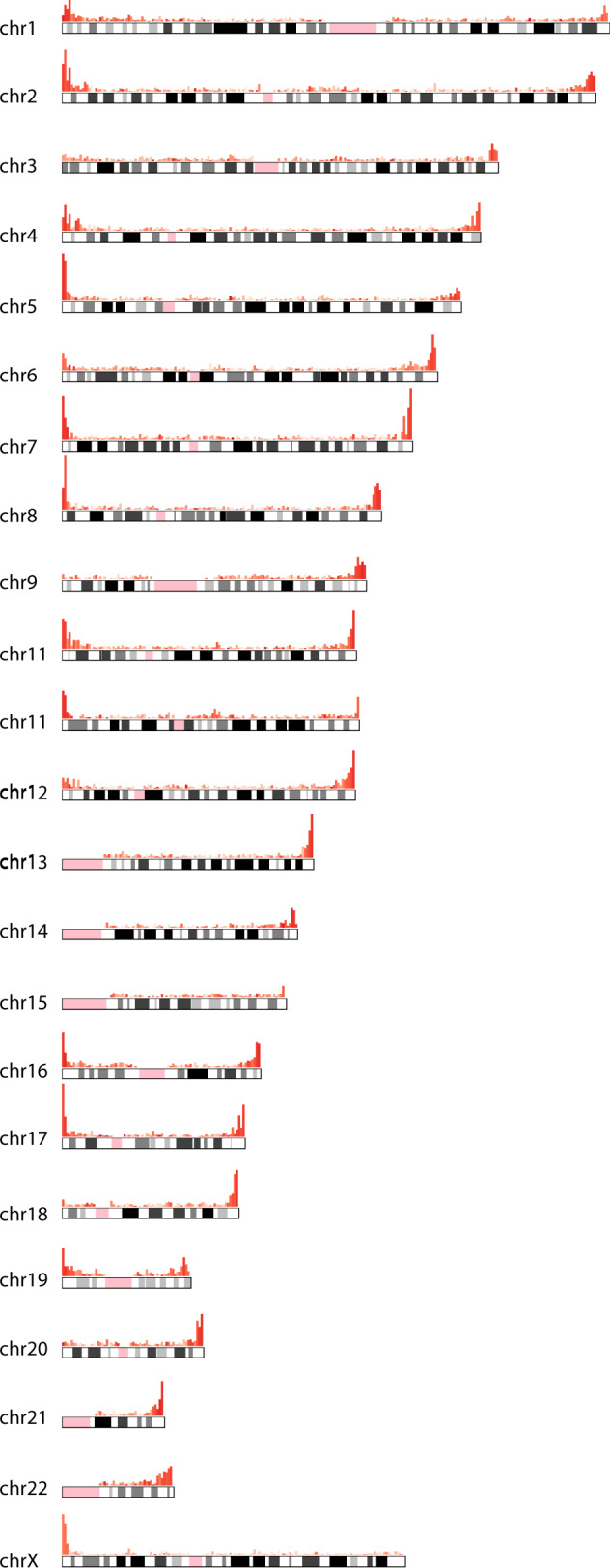
GC-rich VNTRs are more abundant in the subtelomeres (5 Mb before the telomeres). Histograms on top of each chromosome indicate the genomic distribution of VNTRs across the genome. Color intensity indicates the average GC-content in each bin.

**Fig. 4:**
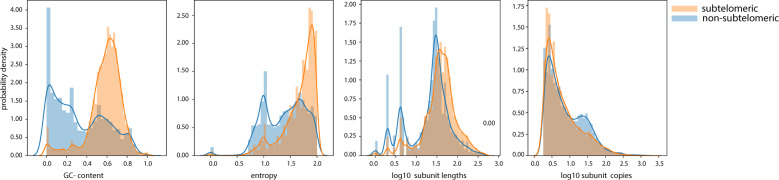
Repeat characteristics are different in subtelomeric VNTRs. Distributions of different VNTR characteristics (as defined by Tandem Repeat Finder) indicate differences between subtelomeric and non-subtelomeric VNTRs. GC-content: the fraction of Guanines (G) and Cytosines (C) in a VNTR; entropy: the quantitative description of the complexity of a VNTR, a measure of sequence conservation and randomness of the VNTR sequence; subunit lengths: a subunit is a stretch of DNA sequence of a given length that is consecutively repeated to form a ‘repetitive sequence’; subunit copies: the number of repetitions of a subunit in a repeat sequence.

The identified SVs were not uniformly distributed across the genome. The SV density in the subtelomeric regions (the last 5 Mb before the telomeres) across all chromosome arms (excluding acrocentric arms) was 5.0-fold higher than the SV density in the non-subtelomeric regions (Fig. 3). We found that the SV enrichment in the subtelomeres was most evident in VNTRs, which were enriched 7.1-fold relative to the genome outside the subtelomeres. Furthermore, we noticed that VNTRs in the subtelomeric regions were more GC-rich and composed of longer repeat patterns than VNTRs outside the subtelomeres (Fig. 4). When we selected VNTRs with a GC-content of more than 60% and repeat patterns longer than 15 bp, we observed an enrichment of 21.1-fold in the subtelomeres (Fig. S2). This did not apply to all subtelomeres as we observed a reduced number of VNTRs on the long arm of chromosome 5, 15, and the X chromosome (specifically the Xq28 region)^30^ as well as the short arms of chromosomes 3, 9, 18, and 20 (Fig. 3).

### Exonic SVs

Of all SVs, 90 mapped within protein-coding exons (~0.3%) (Fig. 2B), as mapped by Gencode (Gencode v30) (Table S3). Many of these exonic SV loci overlapped with previously reported exonic VNTRs (e.g., PER3^31,32^, CEL^33–36^, SAMD1^10,12^, ACAN^37^, and various MUC genes^38,39^). Of these SVs, 17 were unique to the W115 assembly (Table S4). Additionally, we observed 83 SVs that spanned one or more exons (Table S5), of which 16 are unique to the novel W115 assembly (Table S6). SVs that span one or more exons mostly overlap with previously reported copy-number variants (*PCDHA*^40^, *NEB*^41^, *CR1*^42^, *CFHR3/1*^43^, *BTNL8/3*^29^, *OPNMW*^44^, *DEFA1/3*^45^, and others).

### Mobile element insertions (MEI)

**Fig. 5:**
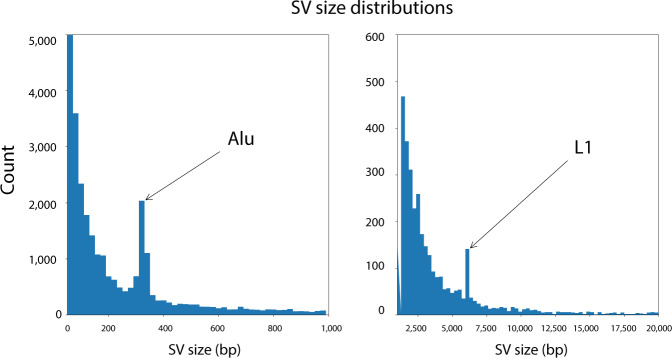
Size distributions of SVs. Size distributions of the SVs indicate peaks at the size of Alu and L1 elements indicating recent insertions of these mobile elements. Left panel: SVs with lengths between 0 and 1000 bp; right panel: SVs with lengths between 1000 and 20,000 bp.

About 10% of the SVs (3005) were insertions of known mobile elements (MEI) (Fig. 2A). The large majority of these insertions (2798, 93%) were Alu transposable elements. Less frequent insertions were due to LINE1/L1 (117, 4%), SVA (73, 2%), and ERV (14, <1%). Of these variants, 737 are unique to the W115 assembly (Table S7). Size distributions of the SVs indicate peaks at the size of Alu and L1 elements indicating recent insertions of these mobile elements (Fig. 5). The actual number of SVs that were caused by MEIs is probably somewhat higher than reported in our analysis, as nested integrations of certain mobile elements were not always classified as such, due to the fact that our approach demanded a reciprocal overlap of at least 80% between the insertion and the documented mobile element sequence (“Methods”).

### Expansion bias in PacBio assemblies with respect to GRCh38

**Fig. 6:**
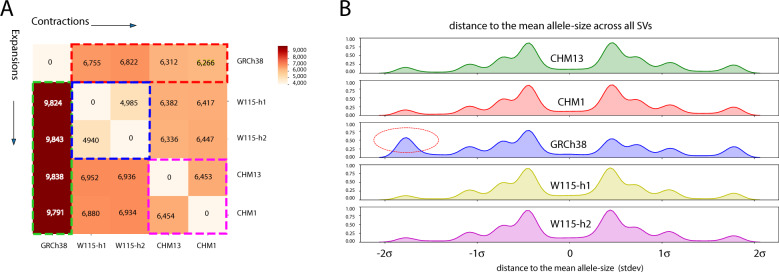
SV alleles are shorter on GRCh38 reference assembly. **A** Pairwise comparison of all SV alleles. Cells on the left of the diagonal display counts of SV alleles for which the haplotype on the *x*-axis is at least 50 bp smaller than the haplotype on the *y*-axis. Cells on the right of the diagonal display counts of SV alleles for which the haplotype on the *x*-axis is at least 50 bp larger than the haplotype on the *y*-axis. All PacBio haplotypes contain more expanded alleles than contracted alleles with respect to GRCh38 (red vs green dotted lines). Expansion vs contraction counts are balanced for CHM1 and CHM13 (blue) and the two w115 haplotypes (pink). **B** Density plots of the difference between SV-lengths to the mean SV-length across all SVs (expressed in standard deviations), per haplotype. Negative values indicate SV alleles with a shorter than average allele (contractions), positive values indicate SV alleles with a longer than average allele (expansions). Red circle: The GRCh38 reference haplotype encodes a disproportionate number of short SV alleles that are ~2 standard deviations smaller than the mean SV allele size. The four PacBio haplotype assemblies show very similar profiles.

We found that ~21% (5978) of the SVs involved expansions with respect to the reference assembly in all four PacBio haplotypes (Fig. 6A). We also observed that SVs were more likely to be expansions (16,974) than contractions (11,948) upon comparing SV alleles of the SMRT assemblies to the GRCh38 reference alleles. This expansion bias was not observed between the CHM1 and CHM13 assemblies and the two haplotypes of the W115 assembly (Fig. 6B). We did observe a slight increase (~1.08-fold) in the fraction of expanded alleles on both W115 haplotypes with respect to the CHM alleles.

### Genes with most VNTRs are predominantly expressed in the brain

**Fig. 7:**
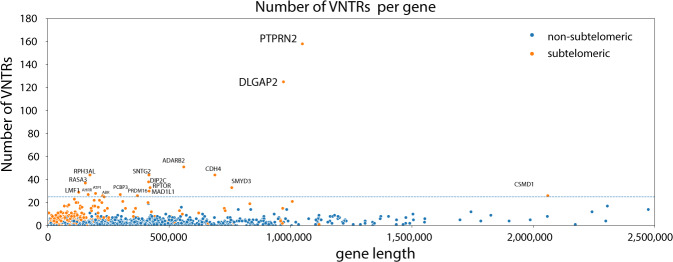
Gene length and position (subtelomeric) influence the number of VNTRs in a gene. Scatterplot of the number of VNTRs that map within a gene. For each gene, the number of VNTRs (vertical axis) are plotted with respect to the length of the gene (horizontal axis). Coloring indicates whether the gene lies within the subtelomeric region (orange) or not (blue). Labeled genes contain more than 25 VNTRs. DLGAP2 and PTPRN2 cover the largest number of VNTRs.

We also investigated whether specific genes were disproportionately affected by structural variation). The top 100 genes that contained the most intronic/exonic VNTRs (Table S8) were enriched for (1) genes that are expressed in the brain (*p* = 1.2e−6, DAVID^46^), (2) genes with multiple splice isoforms (*p* = 1.1e−8, DAVID^46^), and (3) genes involved in autism spectrum disorders (*p* = 6.3e−7, DAVID^46^) (Table S9). However, if we normalize the VNTR count by the length of the gene (Table S10), the observed enrichment of these features is lost (Tables S11). In fact, we observe a correlation between the length of genes and their expression in the brain (see “Methods”, spearman *r* = 0.13, *p* = 7.05e−74)^47,48^, which is stronger than the correlation between the number of VNTRs in a gene and the expression within the brain (see “Methods”, spearman *r* = 0.04, *p* = 1.33e−07). However, there are two genes that contain a disproportionate number of VNTRs which does not seem to be purely related to the length of these genes (Fig. 7). These two genes (DLGAP2 and PTPRN2) are positioned within the subtelomeres of chromosomes 7 and 8, are predominantly expressed in the brain, and were previously associated with a wide range of different neurological phenotypes.

### Inversions

Next, we analyzed the rearrangements between the four assembled haplotypes with a pairwise comparison to the GRCh38 reference assembly (“Methods”). We found that a proper classification of inversions was not trivial (Fig. S3a). Inversions smaller than 100 bp were not always identified as inversions, but as colinear variants. In other cases, inversion events were not completely balanced as they were flanked by large indels (e.g., the event in ATXN2, Fig. S3b) or they were contained within VNTRs. Also, some sequence inversions occurred nested within unstable TA-rich tandem repeats, which, given the similarities to the reverse-complemented sequence, could not be ruled out to be false positives. Across the five haplotypes we detected a total of 162 unique sequence inversions (Fig. S4a and S4b). About 17% (28/162) of the inversions overlapped in all SMRT sequencing assemblies, presumably because the GRCh38 reference assembly was inverted at this position with respect to the other haplotypes. The largest sequence inversion (observed in CHM13) comprised 2.5 Mb of sequence. No inversions were observed within coding sequence or untranslated regions, 103 inversions were observed in intergenic regions, 42 were observed within introns and 17 spanned larger genomic regions that encompassed several genes or coding exons (Tables S12, S13, and S14). In the W115 assembly alone, we detected a total of 104 sequence inversions with respect to the GRCh38 reference genome. Of these inversions, 48 were heterozygous and 56 homozygous. For 42 of these loci, no inversions were observed in the CHM1 or CHM13 assemblies. The largest inversion that was unique to the W115 assembly is 213 kb and spans the entire *COX10* gene (Table S15). When focusing on the X chromosome, we observed that the number of inversions (9) on this chromosome was larger in the CHM1 assembly than the other two assemblies (respectively, 3, 3, and 3 for the W115-1, W115-2, and CHM13 genomes, Fig. S4a). We presumed that this might be due to the increased contiguity of the CHM1 assembly which spans larger regions of the inverted low-copy repeat structures that are characteristic for the X chromosome (Table 1).

### Other rearrangement breakpoints overlap with known segmental duplications

Finally, we characterized the genomic regions of other rearrangement breakpoints and found that 85% overlapped with known segmental duplications. Only one rearrangement event (3 breakpoints) that spans the TNNT3 gene was observed in all assemblies (Table S16) but not in the GRCh38 reference, which appears to have a different arrangement at this locus (Fig. S3c).

## Discussion

In this work, we identified and compared the SVs between five human haplotypes, of which two were contributed by a novel diploid genome of a Dutch woman who reached 115 years with retained cognitive health. We provide an exhaustive compendium of positions and characteristics of the 31,680 SVs across these five genomes. The majority (~70%) of the SVs between these human genomes were VNTRs. Interestingly, we observed that VNTRs in the subtelomeric regions were composed of longer repeat subunits than VNTRs outside the subtelomeric regions, and that they had a higher GC-content. Specifically, with the exception of the subtelomeres of few chromosome arms, the subtelomeric regions of all chromosomes were enriched by a ~21-fold with VNTRs that have >60% GC-content and repeat patterns >15 bp.

### Association of VNTRs with disease risk

The repeat sequences in VNTRs are known to induce alternative secondary structures (such as R-loops^49^), which makes VNTRs vulnerable to mutation^49^. We observed that, as expected, gene length was positively correlated with the number of VNTRs. Furthermore, we found that genes that contained most VNTRs were enriched for genes expressed in the brain, specifically genes with multiple splice isoforms and genes associated with autism spectrum disorders. This may be explained by the observation that long genes are predominantly expressed in brain^47,48^. Therefore, we speculate that VNTRs across the genome may particularly affect neurological function. For example, the two genes that contain the most VNTRs in our analysis, DLGAP2 and PTPRN2, are predominantly expressed in the brain and were previously associated with a wide range of different neurological phenotypes: rare CNVs in DLGAP2 were associated with the autism spectrum^50,51^; rare CNVs in PTPRN2 were associated with attention-deficit hyperactivity disorder^52,53^), GWAS markers in PTPRN2 were associated with schizophrenia/bipolar disorder^54^; rare single-nucleotide variations in DLGAP2 were associated with schizophrenia^55^ and linkage analysis of PTPRN2 gene identified an association with cocaine dependence/depression^56^.

### Variation in subtelomeres

Previous findings indicated that (1) meiotic recombination rates are elevated in the subtelomeres, and (2) that GC-rich repetitive sequence motifs associate with recombination hotspots and genome instability^57–61^. Therefore, the observed inter-individual variability of subtelomeric VNTRs may be caused by faulty meiotic recombination events, while somatic variability between cells may also be induced by homologous recombination that is triggered by erroneous DNA repair (i.e., unequal crossover events and small-scale non-allelic homologous recombination)^8,62–66^. Notably, the recombination rate was previously found to be population specific^67^.

Our findings that GC-rich VNTRs are enriched in the subtelomeres lead us to speculate that evolutionary adaptations are more likely to occur at the subtelomeric ends of chromosomes, while the more conserved central regions within chromosomes have survived evolutionary pressure. Indeed, the subtelomeric enrichment in human genomes of genes that code for highly variable gene families attest to this presumption, with notable examples of the olfactory receptor gene family, the genes encoding immunoglobulin heavy chains, and the zinc-finger protein family^68–71^. The abundance of VNTRs in these regions is an indicator of the ‘evolvability’ of these loci^72–74^. These speculations are supported by findings in yeast where subtelomeric gene families are shown to drive the adaption to environmental changes^75^.

Relative to most autosomal chromosomes, we observed a depletion of SVs in the subtelomeric end of the long arm of the X chromosome, specifically the Xq28 region. This region has been associated with many X-linked diseases^30,76^ and borders the Fragile X locus^76^. This locus encompasses a well-studied VNTR in the 5′-untranslated region of the FMR1 gene, which, when expanded, is the most common cause of mental retardation in males. Together, our observation that VNTRs are not uniformly distributed across the genome and that repeat subunits have specific characteristics, holds potential to model the stability of loci across the genome and to predict the chance that a tandem repeat is a VNTR within a population^77–79^. The dataset presented here can be used to train such models.

### Technical differences between genome assemblies

The W115 genome was assembled from long-read sequences derived from DNA isolated from fresh frozen heart cells, while the CHM1/CHM13 DNA was derived from a molar cell line^17^, and GRCh38 was generated from DNA derived from white blood cells cloned into Bacterial Artificial Chromosomes (BAC) clones so that it could be multiplied and Sanger sequenced. Furthermore, the contiguity of CHM1 was larger compared to the other PacBio haplotypes. Technical differences between the generation of the different assemblies most likely explain the observation that SVs in the PacBio assemblies more often involved expansions compared to contractions when compared to the GRCh38 reference assembly (Fig. 5). It has previously been shown that ‘muted’ gaps^4,10^ and/or unstable genomic sequence causes problems upon cloning, which may have resulted in shortened alleles in the GRCh38 reference genome^16,80^. At this point, we can only speculate to which extent this technical variation explains observed differences.

### Implementation of SV detection in a clinical setting

While a systematic genome-wide association of SVs, and specifically VNTRs, with specific diseases has not yet been performed, we speculate that SVs may in part explain the missing heritability as observed in many GWAS studies (see Box 2). To further investigate the impact of pathogenic VNTR lengths in patients, we suggest that a compendium of non-pathogenic VNTR length-distributions is warranted. This should report an estimated threshold of the number of repeats or total repeat lengths that associates with increased risk for specific diseases. Furthermore, in this work, we did not address the somatic instability of the reported VNTRs. However, several repeat sequences have been reported to somatically expand or contract in different tissues^8,81,82^. Therefore, investigation of VNTRs that somatically expand or contract during a lifetime deserves further attention, specifically in relation to their association with age-related diseases.

BOX 2 SVs may explain part of the missing heritability associated with traits and diseases Genome-wide association studies have identified hundreds of single-nucleotide variants across the human genome that associate with various diseases and traits. Generally, these loci associate with only a marginal increase in the risk of having a trait or develop a disease, such that the aggregate GWAS signals often explain only a fraction of the estimated genetic heritability of the disease. It is clear that common single-nucleotide variants, as assessed in GWAS approaches, often are not the causal variants that biologically causes the associations with a trait or disease^86^. Therefore, it is necessary to search for causative genetic variants that are in linkage with the GWAS signals. Yet, despite great efforts, for many GWAS loci the causative genetic variants remain unclear. One explanation to account for this ‘missing heritability’ is that SVs, and specifically VNTRs have not been systematically interrogated for variants that might explain a GWAS signal. This is regrettable, because SVs account for most varying base pairs (bp) among individual human genomes^28^.

Determining the underlying variation that explain these GWAS signals are key to broaden our understanding of the biological mechanisms underlying various diseases. The abundance of SVs that might be in linkage with other GWAS loci is enormous. A recent example of an SV associated with a GWAS signal is the discovery of a subtelomeric VNTR in an intron of the *ABCA7* gene^5^. The size of the VNTR expansion was found to be in linkage disequilibrium with a SNP that was associated with Alzheimer’s disease in the case of control GWAS studies. This VNTR is one of many intronic SVs detected in this work. Also, various exonic VNTR maps within a very short distance of genome-wide significant loci associated with different GWAS studies. Examples are height (*ACAN* locus^37^), hair morphology (*TCHH* locus^87^), Asthma/Atopy (*FLG* locus^88,89^), Rheumatoid arthritis (*ICOSLG* locus^90^), and diastolic blood pressure (*HRCT1* locus^91^). Future studies will have to indicate whether these VNTRs indeed explain at least part of the disease associations as identified in the respective GWAS studies.

## Conclusions

In-depth assessment and comparison between five human genomes indicated that genetic instability specifically occurs in the subtelomeric ends of chromosomes. We find that these genetic loci, characterized by repeat-sequence variations, represent an important novel layer of genetic variation that should be included in investigations of genetic factors associated with phenotypic traits, specifically those associated with neurological disorders.

## Supplementary information

Supplement

Supplementary Tables

## Data Availability

All data generated or analyzed during this study are included in this published article (and its supplementary information files). All datasets (including raw long and short-read sequencing data) generated and/or analyzed during the current study are available through the European Nucleotide Archive (ENA) and European Variation Archive (EVA) under accession: PRJEB39817. The de novo assemblies are archived under accession numbers: GCA_903995575 (haplotype 1) and GCA_904060995 (haplotype 2).
