# Supplementary material for: Extreme enrichment of VNTR-associated polymorphicity in human subtelomeres: genes with most VNTRs are predominantly expressed in the brain
Source: Transl Psychiatry. 2020 Nov 2;10:369. doi: 10.1038/s41398-020-01060-5 (PMC7608644; doi:10.1038/s41398-020-01060-5)
Supplement: Supplementary file 1 — Supplement [file 41398_2020_1060_MOESM1_ESM.docx]

# Supplementary Data

# **Extreme enrichment of VNTR-associated polymorphicity in human subtelomeres: genes with most VNTRs are predominantly expressed in the brain**

Jasper Linthorst^1,2^, Wim Meert^3^, Matthew S. Hestand^3^, Jonas Korlach^4^, Joris Robert Vermeesch^3^, Marcel Reinders^2^, Henne Holstege^1,2,5*^

Affiliations:

1. Department of Clinical Genetics, Amsterdam Neuroscience, Vrije Universiteit Amsterdam, Amsterdam UMC, Amsterdam, The Netherlands
2. Delft Bioinformatics Lab, Delft University of Technology, Delft, The Netherlands
3. Department of Human Genetics, KU Leuven, Leuven, Belgium
4. Pacific Biosciences, Menlo Park, CA, USA
5. Alzheimer Center Amsterdam, Department of Neurology, Amsterdam Neuroscience, Vrije Universiteit Amsterdam, Amsterdam UMC, Amsterdam, The Netherlands

***Corresponding author:** [h.holstege@amsterdamumc.nl](mailto:h.holstege@amsterdamumc.nl)

Supplementary Figure 1. Pseudo haplotype construction


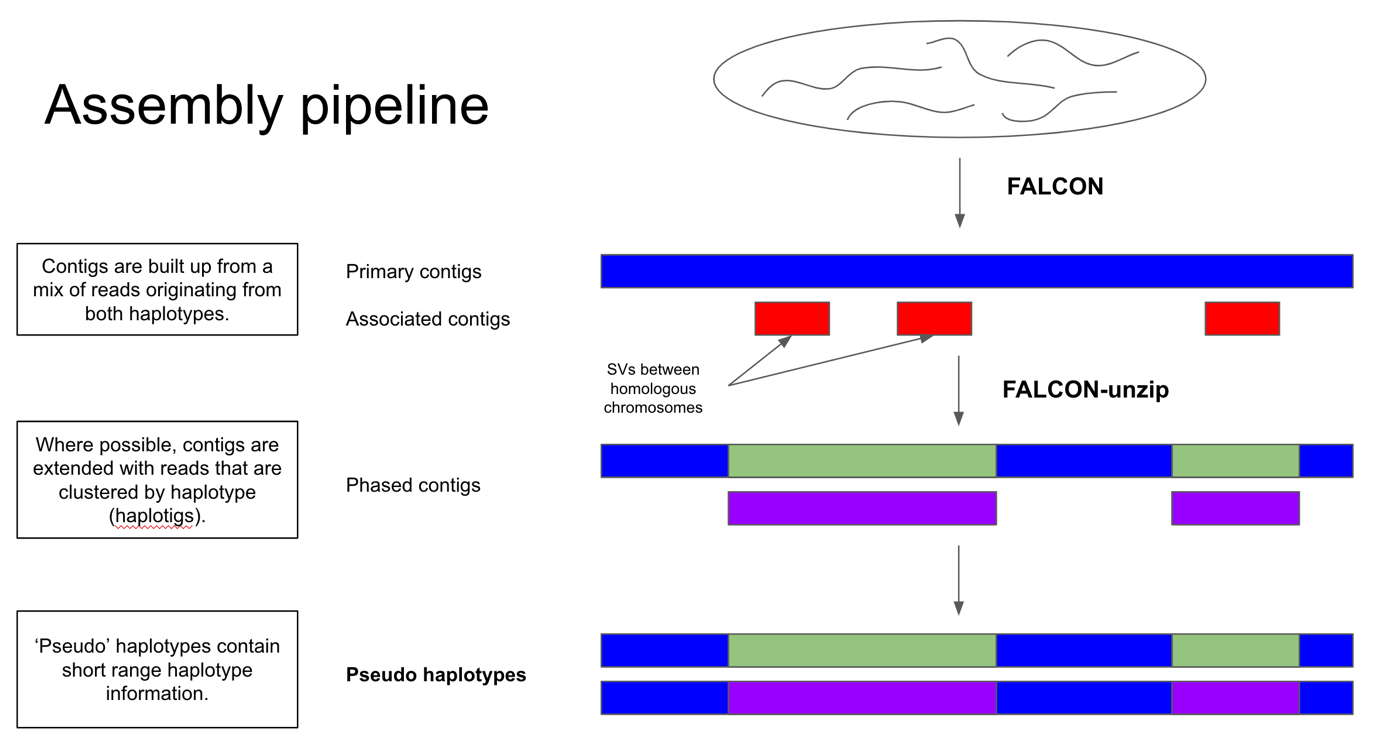


*Schematic representing the construction and definition of pseudo- haplotypes from long read sequencing data.*

## Supplementary Figure 2. Features reported by Tandem Repeat Finder discriminate VNTRs in subtelomeres.


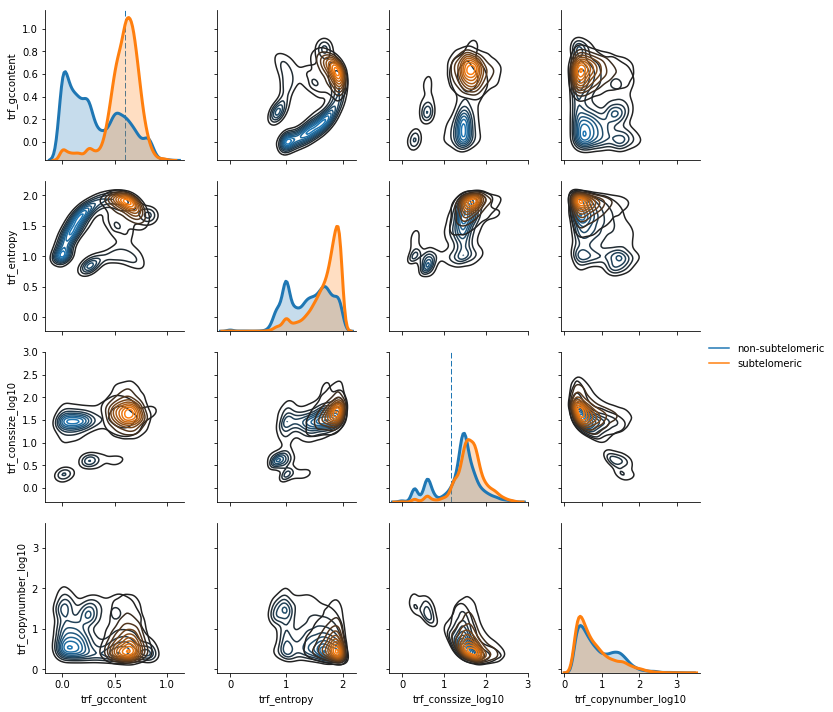


*Density plots of all pairs of features calculated by Tandem Repeat Finder in subtelomeric and non-subtelomeric VNTRs. Dotted blue lines indicate the applied cut-offs for calculating the fold-enrichment of GC-richness (60%) and consensus length (15 bp).*

Supplementary Figure 3a. Complex inversion events


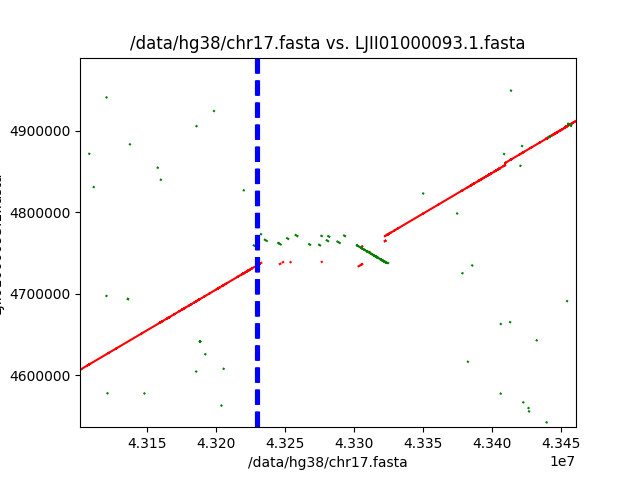

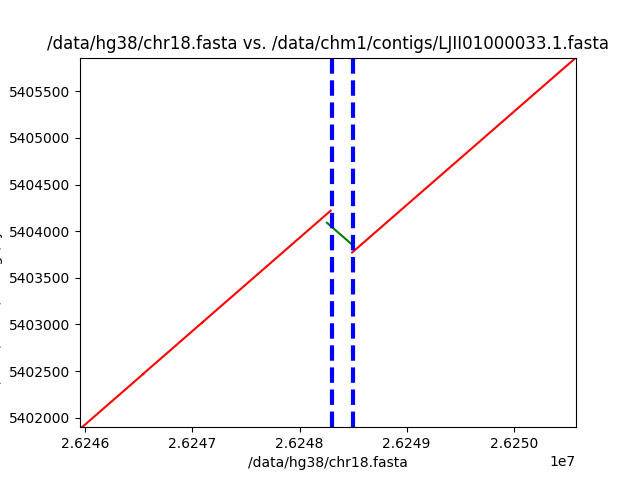


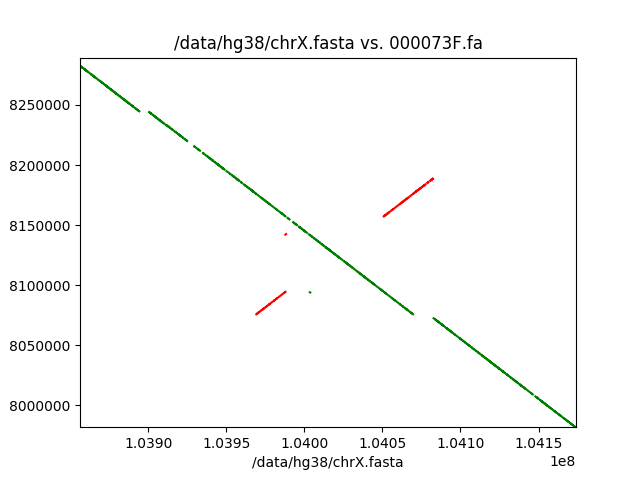

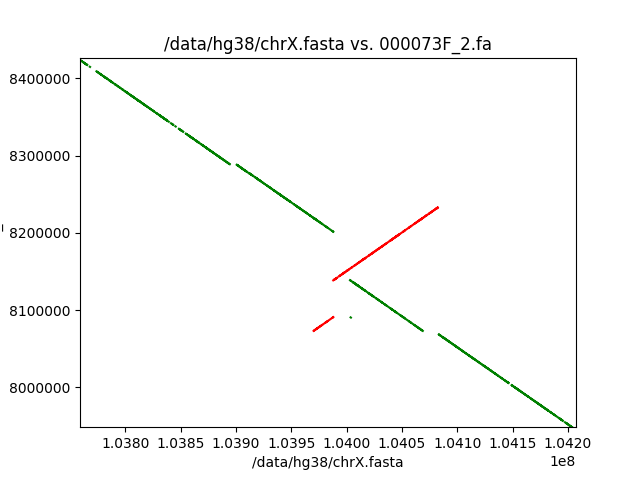


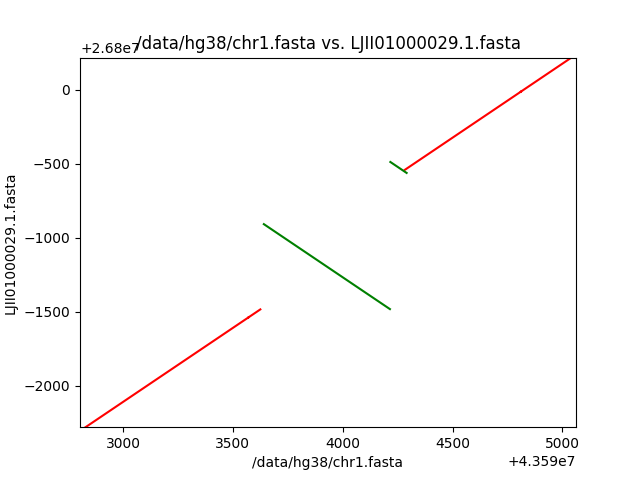


*Various rearrangements across the genome. Red lines indicate clusters of Maximal Unique Matches (MUMs) on the positive strand, while green lines indicate clusters of MUMs on the reverse strand. Many rearrangement events are combinations of events that contain more than just two breakpoints in the alignment.*

## Supplementary Figure 3b. ATXN2 inversion flanked by deletion


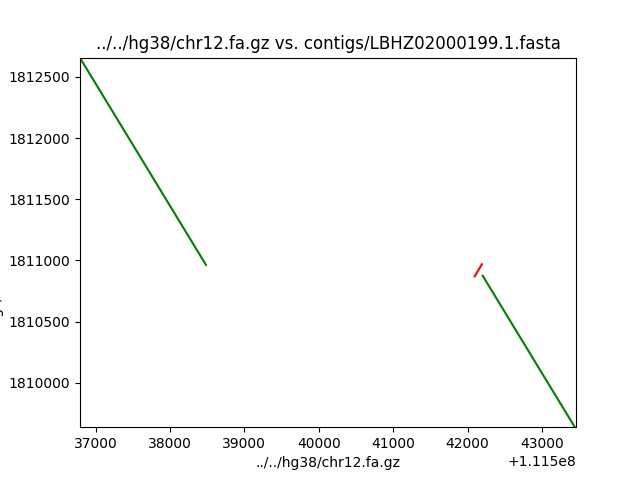


*A rearrangement event in ATXN2 shows an inversion flanked with a deletion in the CHM13 assembly. The same inversion event, but with a smaller deletion was observed in the w115 assembly.*

## Supplementary Figure 3c. Recurrent TNNT3 rearrangement


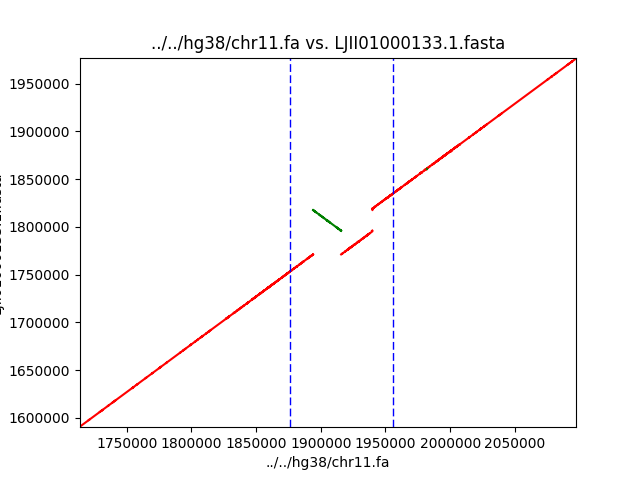

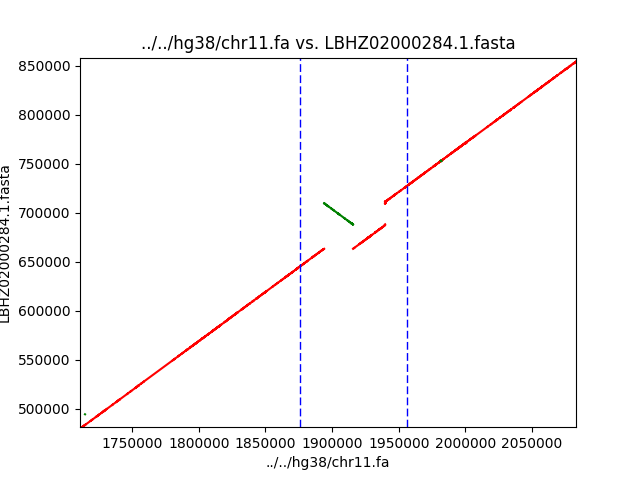

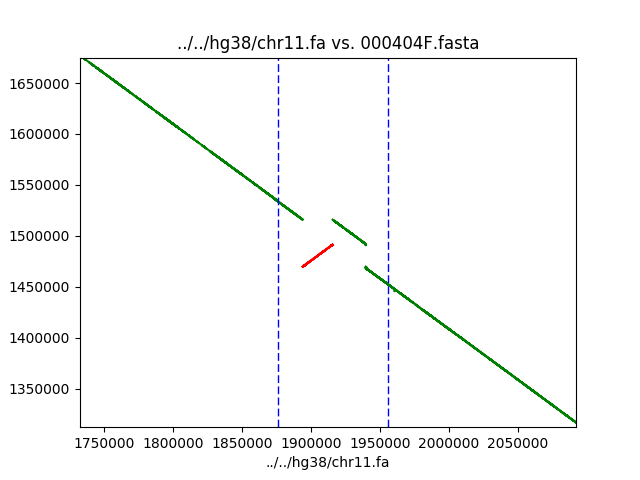

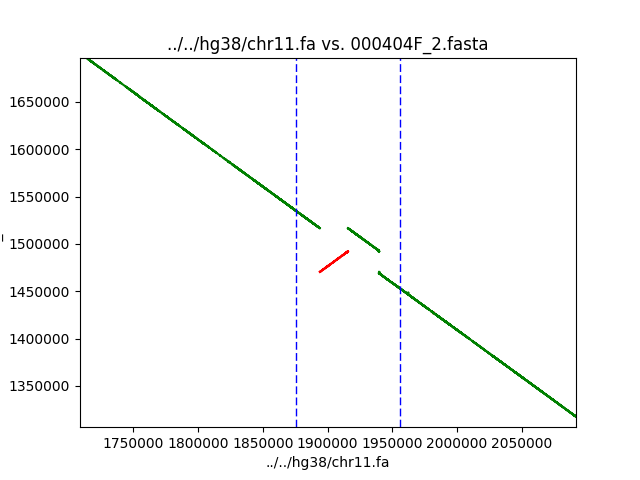


*Many complex rearrangement events appear to be multi-allelic, but the complex rearrangement event in TNNT3 seems to be unique to the GRCh38 assembly.*

## Supplementary Figure 4a. Inversion counts per chromosome


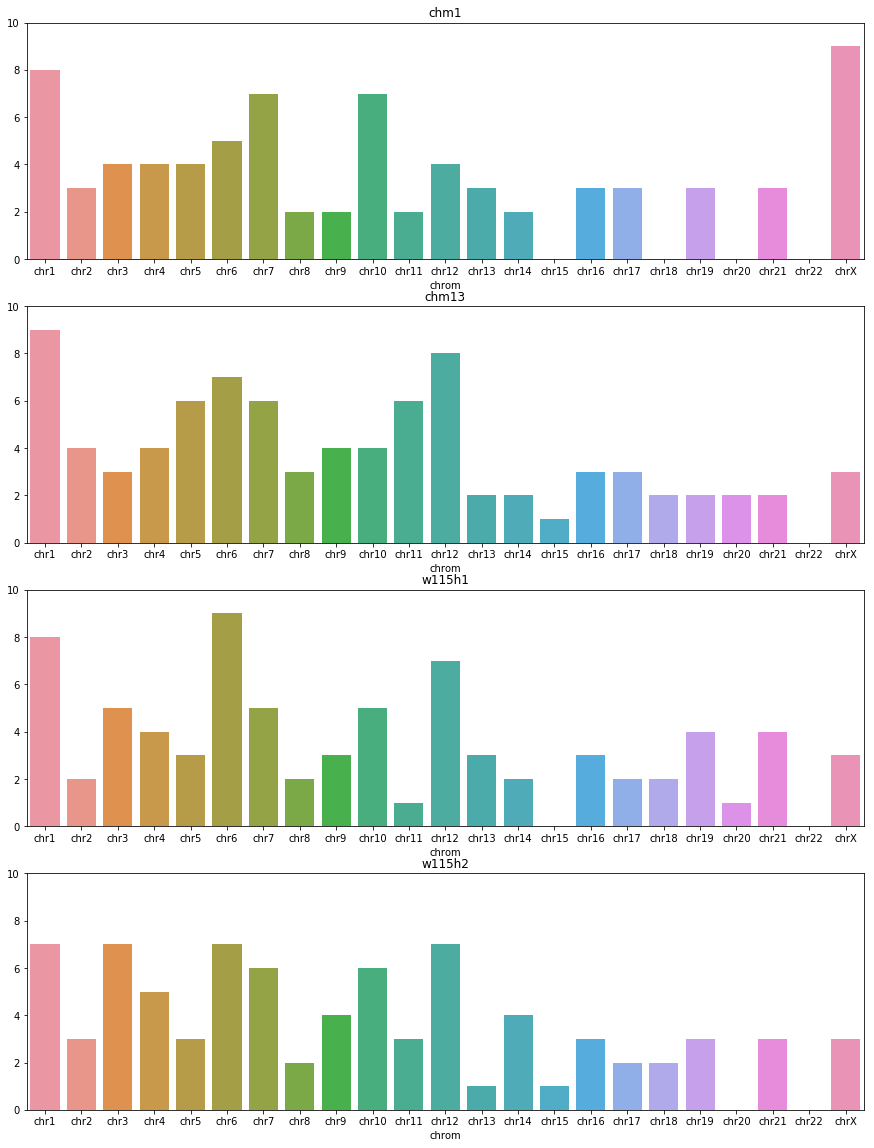


*Distribution of the number of inversions per chromosome and assembly. The CHM1 displays an enrichment of the number of inversion events on the X-chromosome. This can be explained by the fact that the CHM1 assembly is the most contiguous assembly that was compared here.*

## Supplementary Figure 4b. Merged inversion counts per chromosome


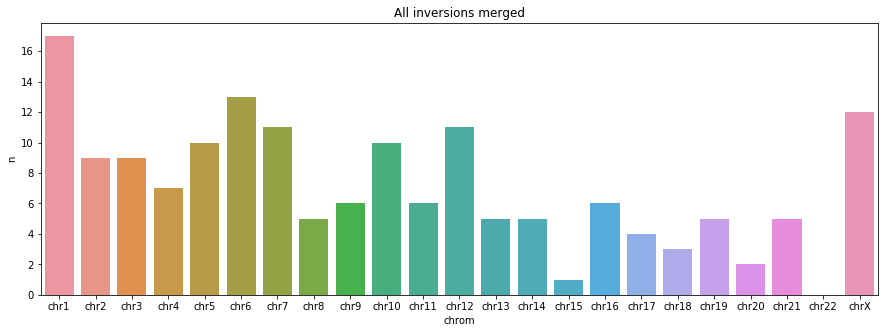


*Distribution of all unique inversion events per chromosome.*
